# Effect of Topical Application of Different Substances on Fibroplasia in Cutaneous Surgical Wounds

**DOI:** 10.5402/2012/282973

**Published:** 2012-02-08

**Authors:** Andreza Miranda Abreu, Dhelfeson Willya Douglas Oliveira, Sandra Aparecida Marinho, Nádia Lages Lima, João Luiz de Miranda, Flaviana Dornela Verli

**Affiliations:** ^1^Nursing Course, Federal University of Jequitinhonha and Mucuri Valleys, 39100-000 Diamantina, MG, Brazil; ^2^Postgraduate Program in Dentistry, Dental Course, Federal University of Jequitinhonha and Mucuri Valleys, 39100-000 Diamantina, MG, Brazil; ^3^Laboratory of Pathology, Department of Basic Sciences, Federal University of Jequitinhonha and Mucuri Valleys, 39100-000 Diamantina, MG, Brazil

## Abstract

*Background*. Fibroblasts on the edges of a surgical wound are induced to synthesize collagen during the healing process which is known as fibroplasia. *Objective*. The aim of this study was to determine the effect of the application of different substances on fibroplasia of cutaneous surgical wounds on rats. *Materials and Methods*. 48 *Wistar* rats were divided into three groups. A surgical wound 1 cm in diameter and 1  mm in depth was created on the dorsum of each animal. The surgical wounds were submitted to the topical application of an alcoholic extract of 30% propolis, 70% alcohol, or 0.001% dexamethasone in a cream base every 12 hours. The animals were sacrificed three, seven, 14, and 28 days postoperatively. The specimens were histologically processed and stained with Masson's trichrome. The assessment of fibroplasia was performed using a scoring system: (1) 5 to 25% collagen deposition; (2) 26 to 50% collagen deposition; (3) 51 to 75% collagen deposition; (4) more than 75% collagen deposition. *Results*. There were statistically significant differences in collagen deposition between the substances at all postoperative evaluation times. *Conclusion*. Propolis and alcohol promoted greater collagen deposition in surgical wounds than dexamethasone.

## 1. Introduction

 The healing process is divided into three phases: inflammatory, proliferative, and remodeling. These distinct, complex phases overlap in order to ensure tissue repair [1–3]. In response to chemotactic factors produced during the inflammatory phase, fibroblasts and endothelial cells are activated to produce collagen fibers and blood vessels, which results in the formation of granulation tissue [4]. The deposition and maturation of collagen fibers in the granulation tissue matrix promotes the formation of conjunctive scar tissue in a process denominated fibroplasia [5–8].

Diverse substances can modulate the different phases of the healing process, including dexamethasone and propolis [7]. Dexamethasone is a synthetic glucocorticoid that promotes a reduction in the proliferation of keratinocytes as well as angiogenesis and fibroplasias in surgical wounds [9]. Propolis is a balsamic resinous substance of a viscous consistency and variable color made by bees (*Apis mellifera*). Its main chemical components include flavonoids, which act as antioxidants, antimicrobial agents, and modulators of the immune system during the healing process [10].

The aim of the present study was to determine the effect of the topical application of propolis, alcohol, and dexamethasone on fibroplasia during the healing of cutaneous surgical wounds on rats through semiquantitative analysis at different postoperative evaluation times.

## 2. Materials and Methods

### 2.1. Sample

This study received approval from the Ethics Committee on Animal Experimentation of the *Universidade Federal dos Vales do Jequitinhonha e Mucuri*, Diamantina, Minas Gerais, Brazil. Forty-eight male rats (*Rattus norvegicus albinus*, Wistar) weighing between 250 and 300 grams and with a mean age of 120 days were kept in plastic cages (60 × 50 × 22 cm) lined with wood shavings and maintained at room temperature. The animals had free access to a balanced chow (Nuvilab, Nuvital Nutrientes SA, Paraná, Brazil) and water.

### 2.2. Surgical Wound

All animals were anesthetized with an intraperitoneal injection of sodium thiopental (Cristália, São Paulo, Brazil, 20 mg/Kg). The hair on the dorsum was cut to skin level in a square shape of approximately 5 × 5 cm with the aid of surgical scissors. A surgical wound 1 cm in diameter and 1 mm in depth was created using a punch.

### 2.3. Experimental Groups

The sample was divided into three groups of 16 animals each. The surgical wounds were submitted to topical applications of different substances every 12 hours: Group 1—alcoholic extract of 30% propolis (Apiário Mackllani Ltda., Santa Bárbara, Minas Gerais, Brazil); Group 2—70% alcohol (Miyako do Brasil Ltda, Guarulhos, São Paulo, Brazil); Group 3—0.001% dexamethasone in a cream base (Belfar Indústria Farmacêutica, Belo Horizonte, Minas Gerais, Brazil).

### 2.4. Sacrifice

The animals were anesthetized with an intraperitoneal injection of sodium thiopental (Cristália, São Paulo, Brazil, 100 mg/kg). The preestablished times for sacrifice were three, seven, 14, and 28 days following the fabrication of the surgical wound in all groups.

### 2.5. Macroscopic Analysis and Histological Staining

The dissected tissue specimens were immersed in a 10% formol solution for fixation for 48 hours. The specimens were examined macroscopically, processed histologically, and stained with Masson's trichrome stain. Collagen fibers exhibit a greenish coloration with the use of this stain.

### 2.6. Analysis of Fibroplasia

**Figure 1:**
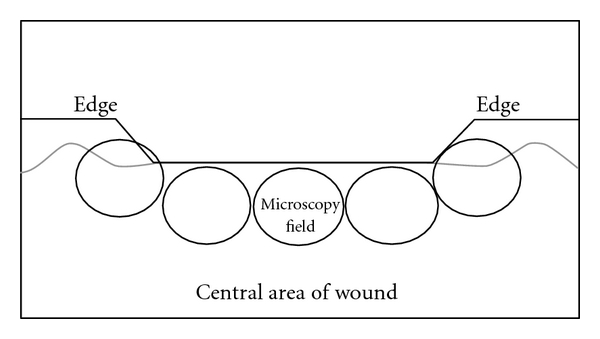
Microscopic analysis of surgical wounds.

**Figure 2:**
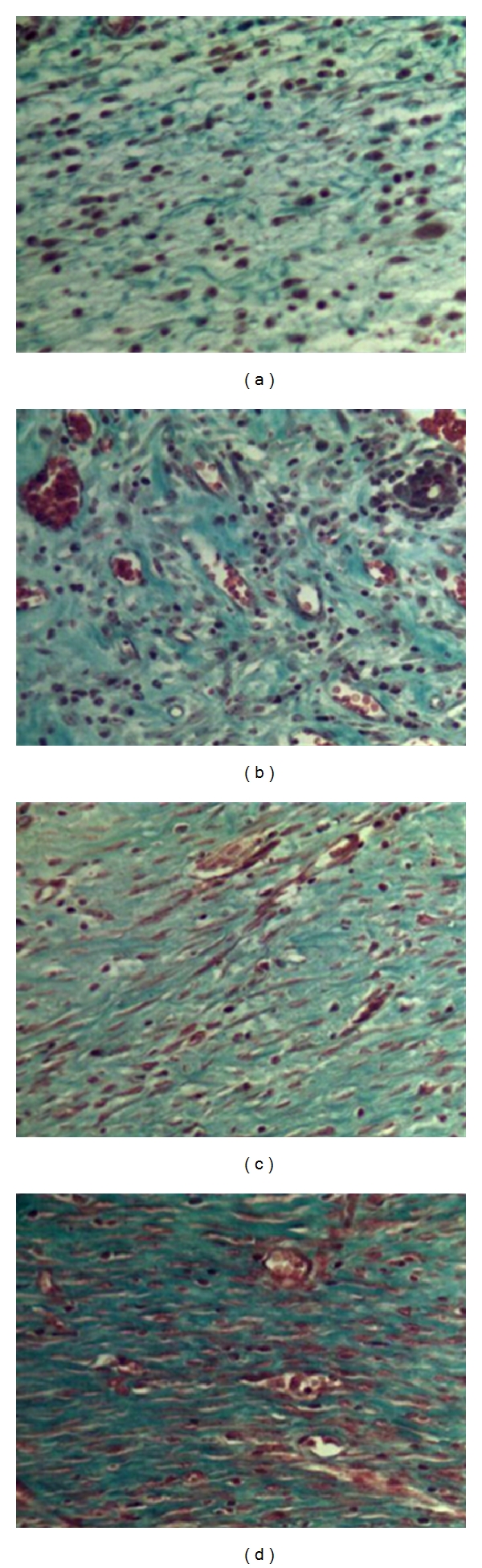
Classification of histological field according to collagen deposition: (a) Score 1; (b) Score 2; (c) Score 3; (d) Score 4 (Masson's trichrome stain, ×200).

For each wound, semi-quantitative analysis was performed in five histological fields—two on the edges and three in the central area of the wound (Figure 1), with 20 fields analyzed per group in each postoperative evaluation time. For such, a light microscope (Olympus BX 41, Japan) was used at a magnification of ×200. The semi-quantitative analysis of fibroplasias was carried out using a scoring system: (1) 5 to 25% collagen deposition, granulation tissue with intense infiltration and a large amount of amorphous substance, (2) 26 to 50% collagen deposition, a reduction in the amount of amorphous substance and the presence of mononuclear inflammatory infiltrate, (3) 51 to 75% collagen deposition, few inflammatory cells between thin, undulated bundles of collagen in the scar tissue, (4) more than 75% collagen deposition, absence of inflammatory infiltrate and presence of dense, parallel bundles of collagen in the scar tissue (Figure 2).

### 2.7. Statistical Analysis

All data collected during the histological analysis were compiled in a data bank using the SPSS program. When appropriate, either the Chi-square test or Fisher's exact test was used to determine significant differences between groups in the comparison of frequencies. The level of significance was set at 5% (*P* < 0.05).

## 3. Results

On Day 3 of the postoperative period, there were statistically significant differences in collagen deposition scores between the propolis and alcohol groups (*P* = 0.013) as well as between the alcohol and dexamethasone groups (*P* = 0.001). All wounds were ulcerated (Table 1). 

On Day 7, there were statistically significant differences in collagen deposition scores between the propolis and alcohol groups (*P* = 0.020), between the alcohol and dexamethasone groups (*P* = 0.001), and between the propolis and dexamethasone groups (*P* = 0.001). There was an increase in collagen deposition in the wounds treated with propolis and alcohol, with partial re-epithelialization of the wound. In the group treated with dexamethasone, all wounds were ulcerated and the collagen deposition score w as 1 (Table 1).

On Day 14, there were statistically significant differences in collagen deposition scores between the propolis and alcohol groups (*P* = 0.014), between the alcohol and dexamethasone groups (*P* = 0.001), and between the propolis and dexamethasone groups (*P* = 0.001). All wounds submitted to either propolis or alcohol were completely re-epithelialized (Table 1). In this time, one Group 1 specimen presented insufficient material for histological analysis and was excluded.

On Day 28, there were statistically significant differences in collagen deposition scores between the alcohol and dexamethasone groups (*P* = 0.001) and between the propolis and dexamethasone groups (*P* = 0.001). All wounds submitted to either propolis or alcohol were completely re-epithelialized, whereas all wounds treated with dexamethasone were partially re-epithelialized (Table 1).

## 4. Discussion

During the healing process, the biosynthesis of collagen by fibroblasts occurs after the migration, activation, and proliferation of these cells for the formation of the granulation tissue, which is a phenomenon known as fibroplasia [11, 12]. In the present study, this process occurred on Days 14 through 28 of the postoperative period in all groups, as evidenced by the greater deposition of collagen. However, each group exhibited different amounts of collagen in the different evaluation times. 

On Day 3, the application of alcohol caused a significantly greater degree of fibroplasia in comparison to dexamethasone and propolis. This may have occurred due to its antiseptic action, which avoided the secondary infection of the surgical wounds, thereby favoring the healing process [13, 14]. According to Percival et al. [15], the presence of bacteria in the region of a surgical wound can lead to an increase in tissue degradation and a delay in the immune response of the host. Moreover, the antimicrobial activity of alcohol is optimal at concentrations between 60 and 90% [14], as demonstrated in the present study using 70% alcohol. 

The collagen fiber bundles were evident on Day 7 of the postoperative period in the propolis and alcohol groups. There was partial re-epithelialization in all the wounds analyzed in these two groups, as collagen deposition in the granulation tissue occurred earlier in comparison to the group having received dexamethasone. Fibroblasts are the main cells involved in fibroplasia and the remodeling of wounds [11]. These cells are normally found in the proliferative and remodeling phase of the healing process and are responsible for the production of collagen and structural extracellular matrix [10]. Assessing the action of orally administered propolis through a nasogastric tube on a colon resection and anastomosis in rats, Kilicoglu et al. [10] report the presence of fibroblasts on the first day of the postoperative period in the group treated with propolis, as compared to the third day in the control group. According to the authors, the fibroblasts that emerged on the first day were more immature cells, whereas those on Day 3 were more mature, with characteristics of active synthesis. The authors also report the presence of collagen fibers on the third day of the postoperative period, unlike what occurred in the present study, in which collagen fibers emerged somewhat later. Moreover, the authors found greater fibroblast proliferation, activation, and synthesis capacity in the group treated with propolis in comparison to the control group, meaning that propolis inhibited the inflammatory response, but stimulated the synthesis of collagen by fibroblasts. In the present study, alcohol promoted a significantly greater degree of fibroplasia in comparison to propolis at seven days following surgery. The likely reason for this is the fact that propolis has greater anti-inflammatory activity due to the presence of flavonoids [16] in the initial inflammation period, which delays the process of fibroplasia. The propolis has the capacity to act in both the acute and chronic phases of inflammation [17]. Two weeks into the postoperative period, collagen reorganization occurs, together with the contraction of the wound [2, 18]. In the present study, this was evident by the greater than 51% collagen deposition in the alcohol and propolis groups and the presence of few inflammatory cells, indicating the onset of the remodeling of the wound. In the propolis group, there was a greater predominance of denser and parallel collagen bundles (type I collagen), suggesting a more advanced remodeling phase in comparison to the alcohol group. The propolis may act by minimizing the acute inflammatory exudate as well as stimulating macrophages and T lymphocytes and, consequently, fibroblast activity [10]. In the present study, this was evident by the reduction in inflammatory cells in the propolis and alcohol groups after 14 days. Moura et al. [7] found that propolis had the capacity to hinder the deposition rate of type I collagen only in the earliest phases of the healing process (Days 4 to 7 of the postoperative period), with collagen deposition reaching the same degree as that in the control group at two weeks. According to the authors, it is likely that the attenuation of the recruitment of cells by propolis accelerated the proliferative phase of the healing process, promoting the rapid transformation of type III collagen into type I and modulating the inflammation process. Beginning at Day 14 of the postoperative period in the present study, propolis also promoted the complete re-epithelialization of the wound. Re-epithelialization is a process that begins within hours following an injury and is finalized when the wound is covered with granulation tissue [11]. Within this same period, re-epithelialization was also complete in the alcohol group, but there was a significantly lesser degree of fibroplasia in comparison to the propolis group. This may be due to the fact that the propolis was an alcoholic extract, thereby exhibiting antiseptic properties along with the known immunomodulating, anti-inflammatory and healing properties of propolis [7, 19, 20], which further accelerate the healing process in wounds. The flavonoids in propolis act as antioxidant, antimicrobial, and immunomodulating agents [10]. Moreover, the alcoholic propolis extract used in the present study has a higher content of flavonoids than the aqueous extract [16].

In the group treated with dexamethasone, inflammatory cells persisted two weeks following the surgery, with less than 50% collagen deposition in the majority of wounds. With the reduction in the proliferation of keratinocytes, angiogenesis, and fibroplasia caused by dexamethasone, there was a delay in the complete re-epithelialization of the wound. Fibroplasia was significantly lower in comparison to that achieved in the other groups at all postoperative evaluation times, with an absence of complete re-epithelialization on Day 28, thereby demonstrating the reduced rate of healing when using dexamethasone. Oishi et al. [9] found that the subcutaneous administration of dexamethasone to wounds on rats affected both the synthesis and degradation of type I collagen in thicker fibers, with greater action on type III collagen, which plays an important role in the onset of the healing process. The degradation of type I and III collagen fibers is carried out by metalloproteinases (MMPs), and the reduction in the level of these collagenases in response to treatment with glucocorticoid must be one of the causes of the delay in the healing process [9]. MMP-8 is a collagenase synthesized by neutrophils that is necessary in order for the healing process to be completed and has the function of the cleavage and denaturation of collagen fibers [21, 22]. In MMP-8-deficient mice, Gutiérrez-Fernández et al. [21] found that re-epithelialization on the third day of the postoperative period was significantly lesser in comparison to mice that synthesized this enzyme, with the wounds completely re-epithelialized by Day 7 in the latter group and only partially re-epithelialized in the group with MMP-8 deficiency. The authors state that this may be due to an apoptosis defect in the neutrophils, as these cells were still present on Days 5 and 7 in the MMP-8-deficient mice. Moreover, normal inflammatory response was reestablished in the MMP-8-deficient group that received a bone marrow transplant, demonstrating the importance of this enzyme in the later phases of tissue repair.

In the present study, the degree of fibroplasia was similar in the groups treated with propolis and alcohol, but significantly lesser in the group treated with dexamethasone. Due to its potent anti-inflammatory action in the early phases of the healing process, dexamethasone has the capacity to reduce the activation, proliferation, and survival of inflammatory cells [23], with a consequent reduction in fibroplasia and re-epithelialization. Nguyen et al. [23] studied the effect of corticoids on inflammatory function in surgical wounds on rats and found a reduction of at least 50% in the circulation of inflammatory cells in the surgical site one day following the procedure, followed by a lesser degree of this reduction on Days 3 and 5.

## 5. Conclusion

Among the different substances applied to surgical wounds in the present study, propolis and alcohol achieved better results than dexamethasone in the promotion of fibroplasia and re-epithelialization, thereby helping to accelerate the healing process.

## Figures and Tables

**Table 1:** Distribution of collagen deposition scores at different postoperative evaluation times.

Postoperative evaluation time	*Group 1*: Propolis *n* (%)	*Group 2*: Alcohol 70% *n* (%)	*Group 3*: Dexamethasone *n* (%)	*P*
*3 days*				*G*1 × *G*2 = 0.013^C^
Score 1	18 (36.7) U	11 (22.4) U	20 (40.8) U	*G*2 × *G*3 = 0.001^F^
Score 2	2 (18.2) U	9 (81.8) U	0 (00.0)	*G*1 × *G*3 = 0.487^F^

*7 days*				*G*1 × *G*2 = 0.020^F^
Score 1	6 (23.1) PR	0 (00.0)	20 (76.9) U	*G*2 × *G*3 ≤ 0.001^C^
Score 2	14 (41.2) PR	20 (58.8) PR	0 (00.0)	*G*1 × *G*3 ≤ 0.001^C^

*14 days*				
Score 1	0 (00.0)	0 (00.0)	11 (100.0) U	*G*1 × *G*2 = 0.014^C^
Score 2	0 (00.0)	0 (00.0)	9 (100.0) U	*G*2 × *G*3 ≤ 0.001^C^
Score 3	5 (25.0) TR*	15 (75.0) TR	0 (00.0)	*G*1 × *G*3 ≤ 0.001^C^
Score 4	10 (66.7) TR*	5 (33.3) TR	0 (00.0)	

*28 days*				
Score 2	0 (00.0)	0 (00.0)	13 (100.0) PR	*G*1 × *G*2 = 1.000
Score 3	0 (00.0)	0 (00.0)	7 (100.0) PR	*G*2 × *G*3 ≤ 0.001^C^
Score 4	20 (50.0) TR	20 (50.0) TR	0 (00.0)	*G*1 × *G*3 ≤ 0.001^C^

^C^chi-square test, ^F^Fisher's extract test, 1.000 = identical groups.

U: ulcerated, PR: Partially re-epithelialized, TR: Totally re-epithelialized.

*It was analyzed fifteen microscopy fields.

## References

[1] Waldorf H, Fewkes J (1995). Wound healing. *Advances in Dermatology*.

[2] Gilmore MA (1991). Phases of wound healing. *Dimensions in Oncology Nursing*.

[3] Aukhil I (2000). Biology of wound healing. *Periodontologyx*.

[4] Greiling D, Clark RAF (1997). Fibronectin provides a conduit for fibroblast transmigration from collagenous stroma into fibrin clot provisional matrix. *Journal of Cell Science*.

[5] Follonier L, Schaub S, Meister JJ, Hinz B (2008). Myofibroblast communication is controlled by intercellular mechanical coupling. *Journal of Cell Science*.

[6] Desmouliere A (1995). Factors influencing myofibroblast differentiation during wound healing and fibrosis. *Cell Biology International*.

[7] Moura SA, Ferreira MA, Andrade SP, Reis ML, Noviello MD, Cara DC (2011). Brazilian green propolis inhibits inflammatory angiogenesis in a murine sponge model. *Evidence-Based Complementary and Alternative Medicine*.

[8] Lee H, Overall CM, McCulloch CA, Sodek J (2006). A critical role for the membrane-type 1 matrix metalloproteinase in collagen phagocytosis. *Molecular Biology of the Cell*.

[9] Oishi Y, Fu ZW, Ohnuki Y, Kato H, Noguchi T (2002). Molecular basis of the alteration in skin collagen metabolism in response to in vivo dexamethasone treatment: effects on the synthesis of collagen type I and III, collagenase, and tissue inhibitors of metalloproteinases. *British Journal of Dermatology*.

[10] Kilicoglu SS, Kilicoglu B, Erdemli E (2008). Ultrastuctural view of colon anastomosis under propolis effect by transmission electron microscopy. *World Journal of Gastroenterology*.

[11] Clark RAF (2001). Fibrin and wound healing. *Annals of the New York Academy of Sciences*.

[12] Clark RAF (1993). Regulation of fibroplasia in cutaneous wound repair. *American Journal of the Medical Sciences*.

[13] Robson MC, Stenberg BD, Heggers JP (1990). Wound healing alterations caused by infection. *Clinics in Plastic Surgery*.

[14] Mcdonnell G, Russell AD (1999). Antiseptics and disinfectants: activity, action, and resistance. *Clinical Microbiology Reviews*.

[15] Percival SL, Thomas JG, Williams DW (2010). Biofilms and bacterial imbalances in chronic wounds: Anti-Koch. *International Wound Journal*.

[16] Schnitzler P, Neuner A, Nolkemper S (2010). Antiviral activity and mode of action of propolis extracts and selected compounds. *Phytotherapy Research*.

[17] Borrelli F, Maffia P, Pinto L (2002). Phytochemical compounds involved in the anti-inflammatory effect of propolis extract. *Fitoterapia*.

[18] Chen WYJ, Abatangelo G (1999). Functions of hyaluronan in wound repair. *Wound Repair and Regeneration*.

[19] Sehn E, Hernandes L, Franco SL, Gonçalves CCM, Baesso ML (2009). Dynamics of reepithelialisation and penetration rate of a bee propolis formulation during cutaneous wounds healing. *Analytica Chimica Acta*.

[20] McLennan SV, Bonner J, Milne S (2008). The anti-inflammatory agent Propolis improves wound healing in a rodent model of experimental diabetes. *Wound Repair and Regeneration*.

[21] Gutiérrez-Fernández A, Inada M, Balbín M (2007). Increased inflammation delays wound healing in mice deficient in collagenase-2 (MMP-8). *FASEB Journal*.

[22] Kondo Y, Fukuda K, Adachi T, Nishida T (2008). Inhibition by a selective I*κ*B kinase-2 inhibitor of interleukin-1-induced collagen degradation by corneal fibroblasts in three-dimensional culture. *Investigative Ophthalmology and Visual Science*.

[23] Nguyen H, Lim J, Dresner ML, Nixon B (1998). Effect of local corticosteroids on early inflammatory function in surgical wound of rats. *Journal of Foot and Ankle Surgery*.

